# Identical *IFT140* Variants Cause Variable Skeletal Ciliopathy Phenotypes—Challenges for the Accurate Diagnosis

**DOI:** 10.3389/fgene.2022.931822

**Published:** 2022-07-07

**Authors:** Joanna Walczak-Sztulpa, Anna Wawrocka, Cenna Doornbos, Ronald van Beek, Anna Sowińska-Seidler, Aleksander Jamsheer, Ewelina Bukowska-Olech, Anna Latos-Bieleńska, Ryszard Grenda, Ernie M. H. F. Bongers, Miriam Schmidts, Ewa Obersztyn, Maciej R. Krawczyński, Machteld M. Oud

**Affiliations:** ^1^ Department of Medical Genetics, Poznan University of Medical Sciences, Poznan, Poland; ^2^ Department of Human Genetics, Radboud University Medical Center, Nijmegen, Netherlands; ^3^ Donders Institute for Brain, Cognition and Behaviour, Radboud University Medical Center, Nijmegen, Netherlands; ^4^ Centers for Medical Genetics, Poznan, Poland; ^5^ Department of Nephrology, Kidney Transplantation and Hypertension, The Children’s Memorial Health Institute, Warsaw, Poland; ^6^ Center for Pediatrics and Adolescent Medicine, University Hospital Freiburg, Freiburg University Faculty of Medicine, Freiburg, Germany; ^7^ CIBSS—Centre for Integrative Biological Signalling Studies, Freiburg University, Freiburg, Germany; ^8^ Department of Medical Genetics, Institute of Mother and Child, Warsaw, Poland

**Keywords:** *IFT140*, skeletal ciliopathy, cilium phenotype, MZSDS-like features, CED-like features

## Abstract

Ciliopathies are rare congenital disorders, caused by defects in the cilium, that cover a broad clinical spectrum. A subgroup of ciliopathies showing significant phenotypic overlap are known as skeletal ciliopathies and include Jeune asphyxiating thoracic dysplasia (JATD), Mainzer-Saldino syndrome (MZSDS), cranioectodermal dysplasia (CED), and short-rib polydactyly (SRP). Ciliopathies are heterogeneous disorders with >187 associated genes, of which some genes are described to cause more than one ciliopathy phenotype. Both the clinical and molecular overlap make accurate diagnosing of these disorders challenging. We describe two unrelated Polish patients presenting with a skeletal ciliopathy who share the same compound heterozygous variants in *IFT140* (NM_014,714.4) r.2765_2768del; p.(Tyr923Leufs*28) and exon 27–30 duplication; p.(Tyr1152_Thr1394dup). Apart from overlapping clinical symptoms the patients also show phenotypic differences; patient 1 showed more resemblance to a Mainzer-Saldino syndrome (MZSDS) phenotype, while patient 2 was more similar to the phenotype of cranioectodermal dysplasia (CED). In addition, functional testing in patient-derived fibroblasts revealed a distinct cilium phenotyps for each patient, and strikingly, the cilium phenotype of CED-like patient 2 resembled that of known CED patients. Besides two variants in *IFT140*, in depth exome analysis of ciliopathy associated genes revealed a likely-pathogenic heterozygous variant in *INTU* for patient 2 that possibly affects the same IFT-A complex to which IFT140 belongs and thereby could add to the phenotype of patient 2. Taken together, by combining genetic data, functional test results, and clinical findings we were able to accurately diagnose patient 1 with “IFT140-related ciliopathy with MZSDS-like features” and patient 2 with “IFT140-related ciliopathy with CED-like features”. This study emphasizes that identical variants in one ciliopathy associated gene can lead to a variable ciliopathy phenotype and that an in depth and integrated analysis of clinical, molecular and functional data is necessary to accurately diagnose ciliopathy patients.

## 1 Introduction

Ciliopathies are a group of rare disorders that show clinical and genetic heterogeneity. The majority of ciliopathies are recessively inherited, however, few autosomal dominant forms (e.g. polycystic kidney disease, ADPKD) and X-linked inheritance patterns (e.g. retinitis pigmentosa, RP) have been reported (Tsang and Sharma, 2018; Ma, 2021). To date 35 ciliopathies and >187 associated disease genes have been described in literature (Reiter and Leroux, 2017). Those ciliopathies presenting with significant skeletal abnormalities are collectively called skeletal ciliopathies, i.e. Jeune asphyxiating thoracic dystrophy (JATD), short-rib polydactyly syndrome (SRPS), Mainzer-Saldino syndrome (MZSDS) and cranioectodermal dysplasia (CED). The skeletal ciliopathy cluster of CED (also called Sensenbrenner syndrome, MIM#218330, MIM#613610, MIM#614099, MIM#614378), MZSDS (MIM#266920, MIM#615630) and JATD (MIM#611263, MIM#613091, MIM#613819, MIM#614376, MIM%208,500) share significant genetic and phenotypic overlap. All three syndromes are characterized by skeletal features; short stature, rhizomelic limb shortening, and narrowing of the thorax to a variable degree where JATD shows the most severe form. A defining feature of JATD and MZSDS is pelvic abnormalities, which distinguishes the two syndromes from CED. While ectodermal abnormalities (i.e. nail, teeth and hair abnormalities) and dysmorphic craniofacial features such as frontal bossing and craniosynostosis are typical for CED and moderately common for MZSDS. The combination of renal and retinal abnormalities is typical for MZSDS, but can also occur in CED and to a much lesser extent in JATD. The major cause of skeletal ciliopathies are defects in genes that encode components of the intraflagellar transport complex A (IFT-A) and complex B (IFT-B). IFT is an evolutionarily conserved bidirectional trafficking machinery that is driven by kinesin- and dynein motor complexes and is essential for proper cilia function and structure. The IFT-B complex is composed of 16 proteins and is responsible for the anterograde transport, while the much smaller IFT-A complex is associated with retrograde transport. The IFT-A complex can be divided into a three core proteins; IFT122, IFT140 and WDR19, and three peripheral proteins; IFT43, WDR35, TTC21B. Dysfunctional IFT140 results in an accumulation of IFT proteins at the ciliary tip and in cilium shortening (Piperno et al., 1998; Miller et al., 2013). Cilia play an important role in diverse signaling pathways including Hedgehog (Hh),Wingless (Wnt), platelet-derived growth factor receptors (PDGFR), mammalian target of rapamycin (mTOR), G-protein coupled receptors (GPCR), Hippo and Notch. These pathways are crucial for normal embryonic development and for tissue homeostasis after birth (Wheway et al., 2019).

Variants in *IFT140* are implicated in the pathogenesis of CED and MZSDS, but also in JATD, Bardet-Biedl syndrome (BBS), Optiz trigonocephaly C syndrome (OTCS), and isolated retinitis pigmentosa (RP) displaying the complexity of ciliopathies (Cole and Snell, 2009; Schmidts et al., 2013; Bifari et al., 2016; Schaefer et al., 2016; Pena-Padilla et al., 2017). Here, we present a clinical, molecular and functional study of two unrelated patients with identical compound heterozygous variants in *IFT140* and show that the observed clinical differences were supported by distinctive cilium phenotypes of the two patients. Moreover, we suggest an alternative description of MZSDS to better resemble the variability seen between patients within this cohort, i.e. “*IFT140*-related ciliopathy with MZSDS- or CED-like features”.

## 2 Materials and Methods

### 2.1 Ethical Considerations

The study was conducted to the ethical tenets of the Declaration of Helsinki and in agreement with the “Code of Conduct for responsible use of patient-derived material”. This study was approved by the Bioethics Committee at Poznan University of Medical Sciences in compliance with The Good Clinical Practice (GCP) and Polish law. Written informed consent was obtained from the patients and their parents.

### 2.2 Collection of Samples

Genomic DNA from both patients and their parents was extracted from whole blood and used for exome sequencing (ES) using a standard method. Skin-derived fibroblasts from both patients were obtained and used for immunofluorescent imaging.

### 2.3 Exome Sequencing

Genomic DNA from the affected patients and the unaffected parents of patient 2 were used for ES . Prior to sequencing, samples were prepared with the Twist library preparation kit (TWIST Bioscience, San Francisco, CA, United States) followed by sequencing on an Illumina Novaseq sequencer (Illumina, San Diego, CA, United States). Reads were aligned to the human genome assembly GRCh37(HG19) using the Burrows-Wheeler aligner version 0.7.13. Variant calling was performed for SNV with GATK HaplotypeCaller version 3.4–46, and for CNV with ExomeDepth version 1.1.12.

### 2.4 *IFT140* Transcript Analysis

RNA was isolated from skin-derived cultured fibroblasts using a standard method. Samples were prepared using the Illumina Stranded mRNA Prep kit according to the manufacturer’s instructions. A total of 18 samples were pooled, equimolar, and sequenced on a SP flowcell using the Illumina NovaSeq6000 (Illumina, San Diego, CA, United States). Demultiplexing and sample analysis with DRAGEN RNA Pipeline 3.7.5 was performed on the Illumina BaseSpace platform.

### 2.5 Cilium Phenotyping Using the Automated ALPACA Tool

The cilium phenotype for both patients was determined using a standardized immunofluorescent assay in skin-derived fibroblasts as described by Doornbos *et al.* (Doornbos et al., 2021). In brief, the fibroblasts were cultured under standard cell culture conditions on glass coverslips. Cilia formation was stimulated by replacement of the culture medium containing 20% fetal calf serum (FCS) to medium containing 0.2% FCS for 48 h prior to staining. Upon fixation, permeabilization and blocking, the cells were stained with antibodies to visualize different cilium parameters, *i.e.* ciliogenesis, cilium length, and retrograde IFT. The ciliogenesis parameter represents the percentage of ciliated cells and is visualized by acetylated-α-tubulin + ARL13B + PCNT. The cilium length was measured using the ALPACA tool, described in detail by Doornbos *et al.*, based on the length of the combined acetylated-α-tubulin and ARL13B signal. The ALPACA tool was also used to measure the surface area of IFT88 and an increased surface area of IFT88 indicates defective retrograde transport. The cilium length and the IFT88 measurement data were combined and plotted into a previously generated reference graph containing data from six control fibroblast lines and ten skeletal ciliopathy patient fibroblast lines (Doornbos et al., 2021).

## 3 Clinical Report

**FIGURE 1:**
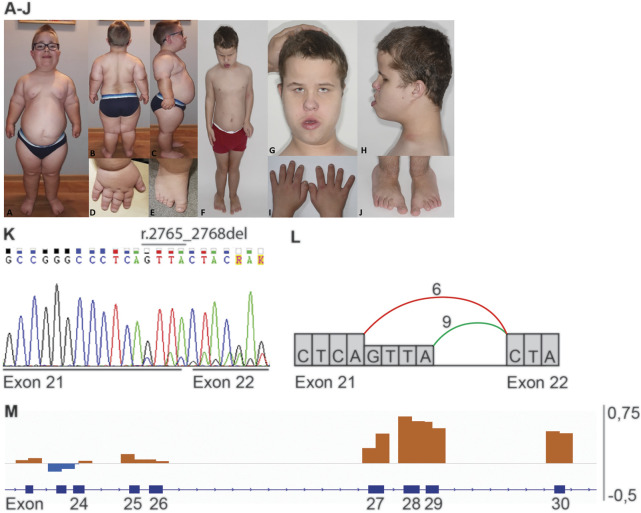
Phenotype and genotype of patients 1 and 2. Proximal limb shortening, narrow thorax, frontal bossing, high forehead, low set and simple ears is present in both patients **(A,F,G,H)**. **(A–E)** Clinical features of patient 1: dolichocephalic head shape, full cheeks, micrognathia **(A)** obesity and hyperlordosis **(B,C)**, brachydactyly and sandal gap **(D,E)**. **(F–J)** Clinical features of patient 2: pectus excavatum **(F)**, epicanthal folds and telecanthus, broad nasal bridge, everted lower lip **(G)**, brachydactyly and sandal gap **(I,J)**. **(K)** Sanger sequence of heterozygous variant r.2765_2768del in *IFT140* (NM_014714.3: c.2767_2768+2del, p.(Tyr923Leufs*28)), representative for patient 1 and 2. **(L)** Schematic representation of the splicing effect caused by IFT140 r.2765_2768del. The coloured lines indicate the splicing between exons, in green the wildtype splicing between exon 21 and 22 and in red the aberrant splicing seen in patients 1 and 2. **(M)** Schematic overview of the heterozygous exon 27–30 duplication (p. (Tyr1152_Thr1394dup)) detected in patients 1 and 2. Orange bars indicate an increase in coverage and blue bars indicate a decrease in coverage.

Detailed clinical characteristics of two unrelated male patients, showing clinical overlap but diagnosed with a different ciliopathy based on differences in the clinical phenotype, presented in Table 1 and Figures 1A–J.

**TABLE 1:** Clinical features of patients with compound heterozygous variants in *IFT140* described in this study and in literature.

Clinical Features	Patient 1	MZSDS^a^	Patient 2	CED^b^; CED^c^
Initial clinical diagnosis	Mainzer-Saldino syndrome (MZSDS)		Cranioectodermal dysplasia (CED)	
Variant protein	p.(Tyr923Leufs*28) ^Pat^ +		p.(Tyr923Leufs*28) ^Pat^ +	
IFT140 NM_014,714.3	p.(Tyr1152_Thr1349dup) ^Mat^		p.(Tyr1152_Thr1349dup) ^Mat^	
Family history	Patient born from the 1^st^ pregnancy		Patient born from the 1^st^ pregnancy. In the 2^nd^ pregnancy the couple had a spontaneous miscarriage at 7^th^ WG. A healthy female was born from the 3^rd^ pregnancy	
Time of delivery	40 WG		39 WG	
Birth measurements				
- weight	- 3720 g (>50^th^ percentile)		- 3410 g (50^th^ percentile)	
- length	- 56 cm (>97^th^ percentile)		- 56 cm (>97^th^ percentile)	
- OFC	- 36 cm (<97^th^ percentile)		- 37 cm (97^th^ percentile)	
Age at examination/current age	10 years/13.5 years		3 months - 13 years/16 years	
Sex	Male		Male	
Dolichocephaly	+	0/6	−	2/2; 7/8
Craniosynostosis	−	3/6	−	1/2; 4/5
Frontal bossing	+	3/3	+	3/3; 4/5
High forehead	+	4/4	+	1/1; NA
Full cheeks	+	3/6	−	3/3; 1/1
Telecanthus/epicanthus	−/+	NA	+/+	NA; 3/4
Broad nasal bridge	−	1/1	+	2/2; NA
Micrognathia	+	NA	−	NA; 2/4
Everted lower lip	−	1/1	+	1/2; 3/4
Low set/simple ears	+/+	2/2	+/+	3/3; 2/2
Narrow chest, pectus excavatum	+, −	7/12, NA	+, +	3/3, 1/2; 10/10, 4/6
Short stature	+	16/21	+	3/3; 4/4
Rhizomelic limb shortening	+	1/7	+	2/2; 8/8
Short ribs	+	2/2	+	2/2; 1/1
Joint laxity	−	NA	+	2/2; 5/5
Brachydactyly of fingers and toes	+	13/15	+	3/3; 11/11
Cone-shaped epiphyses of phalanges	+	17/18	+	3/3; NA
Abnormality of proximal femur	+	1/1	NA	NA; NA
Slender, thin bones	−	0/2	NA	NA; NA
Dental abnormalities	+	0/8	+	3/3; 11/11
- malformed	+		+	
- widely spaced	+		+	
- hypodontia	−		−	
Nail abnormalities	−	NA	+	2/2; 3/4
Thin and/or sparse hair	+/−	NA	−/−	3/3; 6/9
Skin laxity	−	NA	−	NA; 7/8
Inguinal hernias	−	NA	−	NA; 2/2
Kidney disease	+	17/23	+	3/3; 10/11
- chronic renal failure	+		+	
- nephronophthisis	#		−	
- renal cysts	+*		+*	
- sclerosing glomerulopathy	−		+	
Kidney transplantation	+/at 4.5 years of age	5/5	+/at 3 years of age	1/1; 3/3
Ophthalmological problems	+	21/22	+ progressive visual loss (at present time blindness)	3/3; 1/7
- nystagmus	+		+	
- retinal dystrophy	+		+	
- optic nerve atrophy	+ (partial)		+	
Liver disease	−	6/13	−	0/1; 4/10
- hepatic fibrosis	−		−	
- cirrhosis	−		−	
- hepatomegaly	−		−	
Heart defects/cardiac malformations	−/−	NA	−/−	0/3; 1/4
Recurrent respiratory infections	+	2/4	−	3/3; 2/5
Developmental milestones		7/14 delayed		2/3 delayed; 0/4 delayed
- sitting	- 12 months (>99^th^ percentile)		- 36 months (>99^th^ percentile)	
- walking	- 16 months (97^th^ percentile)		- 6 years (>99^th^ percentile)	
- speech development	- 24 months		- absent speech	
Intelligence	Normal	NA	ID	1/3 ID; 0/2 ID
Cerebellar ataxia	+	NA	+	NA
Other findings (CT, MRI, X-rays, EEG etc.)	−		- Congenital CMV infection - Epilepsy (absence attacks) from the age of 13 years, treated with Valproate - MRI - normal	

^a^—IFT140-related MZSDS, phenotype based on literature (Perrault et al., 2012; Schmidts et al., 2013; Geoffroy et al., 2018; Oud et al., 2018); ^b^—IFT140-related CED, phenotype based on literature (Bayat et al., 2017; Walczak-Sztulpa et al., 2020); ^c^—CED, phenotype based on literature, excluding IFT140 (Gilissen et al., 2010; Walczak-Sztulpa et al., 2010; Arts et al., 2011; Bredrup et al., 2011); CT, computed tomography; EEG, electroencephalography; ID, intellectual disability; Mat, maternally inherited variant; MRI, magnetic resonance imaging; NA, data not available; Pat, paternally inherited variant; WG, weeks of gestation. + feature present;—feature absent; *presence, number of renal cysts, revealed by repeated ultrasonography not seen at baseline, has increased over time of follow-up; # unclear.

In summary, patient 1, a 10-year old boy was referred to a clinical geneticist due to severe retinal degradation, facial dysmorphisms, bone dysplasia, skeletal abnormalities and chronic renal failure (after kidney transplantation at 4.5 years of age). Patient 2, a 16-year old boy, presented with facial dysmorphisms, global developmental delay, epilepsy (from 14 years old), abnormal body proportions, skeletal abnormalities, ESRD and congenital CMV infection. The skeletal abnormalities in both patients include narrowing of the chest, short stature, rhizomelic limb shortening, brachydactyly, and cone-shaped epiphyses of phalanges. Besides the significant clinical overlap between patients 1 and 2 there are also typical phenotypic differences, including the presence of dolichocephaly, micrognathia and thin hair in patient 1 but not in patient 2, and, the presence of a broad nasal bridge, everted lower lip, nail abnormalities and joint laxity only in patient 2. In addition, there were striking differences in the development of both patients. Patient 1 showed normal psychomotor development and intelligence, while patient 2 showed developmental delay with intellectual disability and epilepsy. Even though both patients developed early-onset end-stage renal disease (ESRD) the development of the disease was different. Patient 1 developed ESRD at the age of 3 years, while patient 2 was diagnosed with rapid end-stage renal failure due to diffuse glomerulosclerosis and fibrosis at the age of 9 months. Moreover, patient 2 presented with a bilateral vesicoureteral reflux (VUR) grade II.

## 4 Results

### 4.1 ES Revealed Identical Causative Variants in *IFT140*

A targeted analysis was performed selecting exonic and intronic position -20 to +8 variants in 170 ciliopathy associated genes (Radboudumc ciliopathy gene panel version DG3.00 (Radboudumc)) with a frequency <1% in dbSNP151, <5% in in-house db (containing data from >22.000 exomes), and <1% in gnomAD (version 2.1.1). This resulted in 16 variants for patient 1 and 19 variants for patient 2 (Supplementary Tables S1 and S2). CNV analysis was performed by selecting for segments containing any of the 170 ciliopathy associated genes and a frequency <1% in the in-house db. This resulted in two duplications for patient 1 and one duplication for patient 2 (Supplementary Table S3). Subsequently, the combined variants from SNV and CNV were filtered for a recessive inheritance model and a matching phenotype, resulting in a single gene, *IFT140*, in both patients. Both probands carry the same variants in *IFT140* Chr16 (GRCh37):g.1575886_1575889del; NM_014,714.4: r.2765_2768del; p.(Tyr923Leufs*28) and exon 27–30 duplication; p.(Tyr1152_Thr1349dup) (Figure 1K-M). Segregation analysis in both families fit with a recessive inheritance pattern. The deletion variant in *IFT140* r.2765_2768 has previously been reported as a likely pathogenic variant in ClinVar (VCV000863072.3) in a patient with retinal dystrophy, as well as, a patient with MZSDS. The duplication variant in *IFT140* NC_000016.9:g.1568118(NM_014714.4):c (4182 + 99)_(3558)dup is absent from ClinVar, but has been detected in two Polish skeletal ciliopathy patients and in eight families reported by Geoffroy (Geoffroy et al., 2018; Walczak-Sztulpa et al., 2020). These include six families (seven patients) diagnosed with MZSDS, one patient with JATD and one affected individual with features of CED.

A larger duplication, including the variant detected in patients 1 and 2, has been reported once in a patient with a skeletal ciliopathy (VCV000523177.1). In addition to the variants in *IFT140*, a heterozygous variant c.1354G > A; p.Ala452Thr in *INTU* (NM_015693.3) was detected in ES data from patient 2. The minor allele frequency of *INTU* variant c.1354G > A is 0.246% in GnomAD v2.1. 1 (accessed on 19 May 2022). The detected variant was previously described as likely pathogenic in a patient with nephronophthisis and growth retardation by Toriyama *et al.* (Toriyama et al., 2016). Furthermore, the molecular data from patient 2 was analyzed for rare variants (same filters applied as described above) in genes associated with intellectual disability (ID) (Radboudumc ID gene panel version DG3.00 (Radboudumc, 2021)) and analyzed for ID-associated CNVs. The resulting variant list was filtered for both autosomal recessive and autosomal dominant inheritance models and subsequently checked for phenotypic overlap. This approach did not lead to candidate variants that could explain the ID phenotype seen in patient 2.

### 4.2 Aberrant Splicing of *IFT140* Transcript

RNA isolated from skin-derived fibroblasts from patients 1 and 2 was used for *IFT140* transcript analysis. Compared to the reference fibroblast sample both patient samples showed aberrant splicing at the splice donor site of exon 21 where ES revealed a 4bp deletion (Figure 1L). Approximately half of the reads originated from position r.2764 indicating a 4bp deletion r.2765_2768del and would result in a frameshift.

### 4.3 Different Cilium Phenotype Between Patients 1 and 2

**FIGURE 2:**
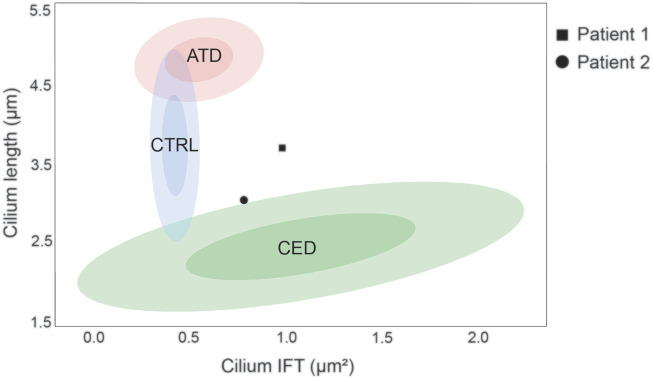
Ciliopathy cilium phenotype clusters. The cilium phenotype clusters are based on two cilium parameters; cilium length (*Y*-axis) and IFT88 measurement (*X*-axis) published in Doornbos *et al.* (Doornbos et al., 2021). The confidence intervals (CI) of 0.5 and 0.9 are indicated per identifiable group, i.e. the control, ATD, and CED cohorts. The cilium phenotype of patient 1 showed a normal cilium length (3.71 ± 0.07 µm) and an increased IFT88 measurement (0.99 ± 0.08µm^2^), therefore it does not fit in any cluster. Patient 2 showed a decreased cilium length (3.04 ± 0.15 µm) and an increased IFT88 measurement (0.79 ± 0.08µm^2^), therefore it is positioned on the border of the CED cluster.

Skin-derived fibroblasts from patients 1 and 2 were used to determine the cilium phenotype based on three parameters; ciliogenesis, cilium length, and retrograde IFT (Supplementary Figure S1). The results were compared to the “healthy cilium phenotype” and that of two distinct skeletal ciliopathy cohorts, ATD and CED, described by Doornbos *et al.* (Table 2) (Doornbos et al., 2021). Fibroblasts from both patients showed normal ciliogenesis >90% and a significantly increased IFT88 measurement of 0.99 ± 0.08µm^2^ for patient 1 and 0.79 ± 0.08µm^2^ for patient 2. While the cilium length for patient 1 was, although slightly increased, within normal range (3.71 ± 0.07 µm), the cilia of patient 2 were significantly shorter (3.04 ± 0.15 µm) (Figure 2). Based on these cilium length and IFT88 measurements, the cilium phenotype of patient 2 resembles that seen in CED patients whereas, the results of patient 1 are clearly abnormal, the cilium phenotype does not cluster with ATD nor CED.

**TABLE 2:** Cilium phenotypes. The cilium phenotypes of three clusters; control, Jeune asphyxiating thoracic dysplasia and cranioectodermal dysplasia published by Doornbos et al. (Doornbos et al., 2021). Followed by the measurements from this study, a control line, patient 1 and patient 2. The cilium phenotypes are represented by the ciliogenesis, cilium length and IFT-A (the IFT88 measurement along the ciliary axoneme).

Group	Ciliogenesis	Length	IFT-A
Controls (*n* = 6)	90 ± 8%	3.68 ± 0.04 µm	0.43 ± 0.01 µm^2^
ATD (*n* = 5)	93 ± 6%	4.81 ± 0.06 µm	0.54 ± 0.03 µm^2^
CED (*n* = 3)	80 ± 14%	2.44 ± 0.05 µm	0.82 ± 0.03 µm^2^
Control 1	91 ± 1%	3.77 ± 0.23 µm	0.47 ± 0.05 µm^2^
Patient 1	93 ± 3%	3.71 ± 0.07 µm	0.99 ± 0.08 µm^2^
Patient 2	90 ± 3%	3.04 ± 0.15 µm	0.79 ± 0.08 µm^2^

## 5 Discussion

The two patients described in this report share identical compound heterozygous variants in *IFT140*. Despite the large phenotypic overlap, the observed clinical differences between patients 1 and 2 led to the suspicion of two different diagnoses. Functional experiments were requested to further investigate the clinical variability, and indeed, the different cilium phenotypes of each patient emphasized the observed clinical variability. The cilium phenotyping data placed patient 2 (decreased cilium length and increased IFT88 measurement) on the border of the established CED cluster, whereas patient 1 (normal cilium length and increased IFT88 measurement) did not fit in any previously defined cluster. Additional experiments are required to further elucidate the exact underlying mechanism that caused this difference in cilium phenotype.

One explanation for the cilium phenotypic difference could be the effect of the likely pathogenic variant found in *INTU* in the ES of patient 2, which was not present in patient 1. Interestingly, INTU has been described to interact with the IFT-A protein complex (Toriyama et al., 2016). Protein interaction studies showed that the ciliogenesis and planar polarity effector (CPLANE) complex proteins, consisting of INTU, FUZ, and WDPCP, interact with all six components of the retrograde IFT complex. The absence of CPLANE inhibits peripheral IFT proteins (IFT43, WDR35, and TTC21B) to localize to the basal body of the cilium and therefore do not assemble onto the IFT core proteins (IFT122, IFT140, WDR19). These data show that there is a connection between INTU and IFT-A components. Therefore, we can speculate that the detected *INTU* variant adds to the burden of the *IFT140* variants on proper functioning of the IFT-A complex. Recent literature has shown that mutations in *INTU* are causative for a short-rib polydactyly syndrome phenotype (Toriyama et al., 2016; Bruel et al., 2018). The *INTU* variant c.1354G > A that was detected in patient 2 has previously been described to be causative in a 13-year old female with nephronophthisis, ESRD at 10 years of age and growth retardation. The authors suggest that the variant may be hypomorphic since the affected residue is poorly conserved and the patient has a milder phenotype compared to other *INTU* patients. It is possible that the likely pathogenic *INTU* variant contributes to the more severe phenotype seen in patient 2 presented in this study, however, further functional studies are required to provide more evidence.

The heterogeneity of ciliopathies is well known. With over 187 associated genes and a pleiotropy of ciliopathy genes for which one gene can cause different phenotypes (Reiter and Leroux, 2017). One example is *WDR19* which is associated with JATD, CED, NPHP, RP, and Senior Løken syndrome (Bredrup et al., 2011; Coussa et al., 2013). In this report, we present two patients with identical compound heterozygous variants and a variable clinical phenotype. A similar case to what we found with the *INTU* variant, was presented by Maglic *et al.* who described two families with variants in *TMEM231* (Maglic et al., 2016). In these two families, of whom the fathers are identical twins, both had children affected with a ciliopathy caused by compound heterozygous variants in *TMEM231*. The mothers carried two different missense variants in *TMEM231* and the fathers carried the same variant. Family 1 had four children with Joubert syndrome (MIM#614970) of which three of the children had an identical phenotype whereas the fourth child was more severely affected. They suggest that an additional variant in the ciliopathy gene *BBS10,* only present in the fourth child, could explain the more severe phenotype. Moreover, the child in the second family presented with Meckel-Gruber syndrome (MIM#615397). Although the compound heterozygous variants between the two families are not identical, it is intriguing to find two different diagnoses in these two closely related families.

We cannot exclude that the developmental delay, ID and epilepsy are a result of the congenital CMV infection that patient 2 had. Recent literature showed that younger children (age 0–2) have an increased risk of developing epilepsy upon experiencing a congenital CMV infection (Lin et al., 2021).

While JATD and CED have cardinal features distinguishing one from the other, this is less clear for MZSDS as we showed in this study. Both presented patients, as well as, published patients with causative variants in *IFT140* display a variable phenotype sharing features with both JATD and CED. This may indicate that the classic description of the skeletal ciliopathy cluster, CED, MZSDS and JATD, does not adequately cover the observed variability within each cohort. Instead “IFT140-related ciliopathy with MZSDS- and/or CED-like features” could be considered to better represent the phenotype of the patient. In our study, the CED-like features such as the ectodermal- and craniofacial dysmorphisms were more prominent in patient 2 compared to patient 1, and together with the suggestive cilium phenotype results we would consider two different diagnoses for each patient. Patient 1 was diagnosed with “IFT140-related ciliopathy with MZSDS-like features” and patient 2 with “IFT140-related ciliopathy with CED-like features”. The intra- and interfamilial clinical variability in patients with identical casual variants has been reported in ciliopathy patients. It is of note that the presence of modifying alleles, environmental factors, and other mechanisms such as epigenetics may play a role in the phenotypic variability. However, it is difficult to precisely define the impact of these factors on the clinical manifestation (Mitchison and Valente, 2017; Walczak-Sztulpa et al., 2021).

The definition of a diagnosis is not set in stone and calls for careful consideration of different facets of the disease. The initial clinical examination, often the guideline for ES analysis, will determine the course of the care path followed by the patient. In our experience the clinical symptoms are leading in defining the differential diagnosis for a patient, but we strongly believe that the addition and integration of molecular genetics data and functional work to the clinical phenotype is crucial to come to an accurate diagnosis for each patient. We showed that the observed clinical differences between two skeletal ciliopathy patients carrying identical causative variants were supported by different cilium phenotypes. Moreover, we suggest to describe the presented phenotypes as “IFT140-related ciliopathies with MZSDS- or CED-like features” to better represent a variable disease cohort. We advocate the necessity of combining clinical, molecular, and functional data to accurately diagnose ciliopathy patients.

## Data Availability

The datasets for this article are not publicly available due to concerns regarding participant/patient anonymity. Requests to access the datasets should be directed to the corresponding author.
